# *In Vivo* Imaging of the Segregation of the 2 Chromosomes and the Cell Division Proteins of *Rhodobacter sphaeroides* Reveals an Unexpected Role for MipZ

**DOI:** 10.1128/mBio.02515-18

**Published:** 2019-01-02

**Authors:** Nelly Dubarry, Clare R. Willis, Graeme Ball, Christian Lesterlin, Judith P. Armitage

**Affiliations:** a Department of Biochemistry, University of Oxford, Oxford, United Kingdom; Institut Pasteur; Max Planck Institute for Terrestrial Microbiology; University of Washington

**Keywords:** MipZ, *Rhodobacter*, cell division, chromosome organization, chromosome segregation, FtsZ

## Abstract

Coordinating chromosome duplication and segregation with cell division is clearly critical for bacterial species with one chromosome. The precise choreography required is even more complex in species with more than one chromosome. The alpha subgroup of bacteria contains not only one of the best-studied bacterial species, Caulobacter crescentus, but also several species with more than one chromosome. Rhodobacter sphaeroides is an alphaproteobacterium with two chromosomes, but, unlike C. crescentus, it divides symmetrically rather than buds and lacks the complex CtrA-dependent control mechanism. By examining the Ori and Ter regions of both chromosomes and associated ParA and ParB proteins relative to cell division proteins FtsZ and MipZ, we have identified a different pattern of chromosome segregation and cell division. The pattern of chromosome duplication and segregation resembles that of Vibrio cholerae, not that of Agrobacterium tumefaciens, with duplication of the origin and terminus regions of chromosome 2 controlled by chromosome 1. Key proteins are localized to different sites compared to C. crescentus. OriC1 and ParB1 are localized to the old pole, while MipZ and FtsZ localize to the new pole. Movement of ParB1 to the new pole following chromosome duplication releases FtsZ, which forms a ring at midcell, but, unlike reports for other species, MipZ monomers do not form a gradient but oscillate between poles, with the nucleotide-bound monomer and the dimer localizing to midcell. MipZ dimers form a single ring (with a smaller diameter) close to the FtsZ ring at midcell and constrict with the FtsZ ring. Overproduction of the dimer form results in filamentation, suggesting that MipZ dimers are regulating FtsZ activity and thus septation. This is an unexpected role for MipZ and provides a new model for the integration of chromosome segregation and cell division.

## Abstract (precis)

Cell division has to be coordinated with chromosome segregation to ensure the stable inheritance of genetic information. We investigated this coordination in the multichromosome bacterium Rhodobacter sphaeroides. By examining the origin and terminus regions of the two chromosomes, the ParA-like ATPase MipZ and FtsZ, we showed that chromosome 1 appears to be the “master” chromosome connecting DNA segregation and cell division, with MipZ being critical for coordination. MipZ shows an unexpected localization pattern, with MipZ monomers interacting with ParB of the chromosome 1 at the cell poles whereas MipZ dimers colocalize with FtsZ at midcell during constriction, both forming dynamic rings. These data suggest that MipZ has roles in R. sphaeroides in both controlling septation and coordinating chromosome segregation with cell division.

## INTRODUCTION

Cell viability relies on mechanisms ensuring accurate cell division. Not only does division usually have to take place at midcell, it has to happen after segregation of the chromosomes. The tubulin-like protein FtsZ is the major division effector recruited to the division plane, where it polymerizes as a dynamic ring intimately involved in the constriction process (1–4).

Division control is based on a balance between positive and negative regulations of the stability of FtsZ polymers. Positive regulators are mainly involved in stabilizing the forming ring, whereas temporal and spatial control is performed by negative regulators, including the Min or MipZ system (5). Recently, additional proteins involved in FtsZ recruitment to midcell, including PomZ (6), SsgAB (7), and MapZ (8, 9), have been identified, suggesting that the mechanism may, however, be more complex and diverse.

The Min system prevents the FtsZ ring forming at sites other than midcell in many species (10–12). For example, in Escherichia coli, ParA-like ATPase MinD recruits MinC, the inhibitor of FtsZ ring formation, and oscillates between poles, with the midcell becoming a MinCD-free zone as the cell grows, allowing the formation of the division complex. In contrast, in Bacillus subtilis, the MinCD proteins localize to the poles and to two rings flanking FtsZ at midcell (13, 14), inhibiting FtsZ ring mispositioning. In both E. coli and B. subtilis, the Min system is complemented by a nucleoid occlusion system (15, 16), allowing coordination of chromosome segregation and cell division.

In the alphaproteobacterium Caulobacter crescentus, a single ParA protein, MipZ, undertakes both of these levels of control (17, 18). MipZ is a direct inhibitor of FtsZ polymerization. It binds the ParB-*parS* DNA complex at the origin (*Ori*) region of the chromosome and forms a gradient from the origin to the new pole. The segregation of the origin regions leads to the establishment of a bipolar gradient of MipZ, preventing FtsZ ring formation other than at midcell. Mechanistically, the gradient formation is based on ParB stimulating the turnover of the MipZ monomers into dimers which inhibit FtsZ polymerization.

The descriptions of the mechanisms presented above are based on species with one chromosome; however, around 10% of sequenced bacteria have a multipartite genome. While the physiology of these species has often been studied in detail, little is known about the behavior of the multipartite genomes throughout the cell cycle (19–22). Rhodobacter sphaeroides is an alphaproteobacterium that is mainly studied for its physiological versatility (23, 24), but it was also the first bacterium with a composite genome to be identified (25). Its genome has two circular chromosomes of ∼3 (C1) and ∼1 (C2) Mb. Here, using cell biology and fluorescence microscopy, we show that the segregation pattern for the origin (*OriC*) and terminal (*Ter*) loci of these chromosomes is different from the patterns seen with the multichromosomic alphaproteobacteria studied to date (19, 22). We also show that a MipZ homologue not only is involved in coordinating the segregation of C1 to cell division but also localizes to midcell in a pattern very different from that characterized in C. crescentus, suggesting a previously unidentified role for this regulator in cell division and control of FtsZ.

## RESULTS

### A MipZ homologue controls cell division in R. sphaeroides.

**FIG 1:**
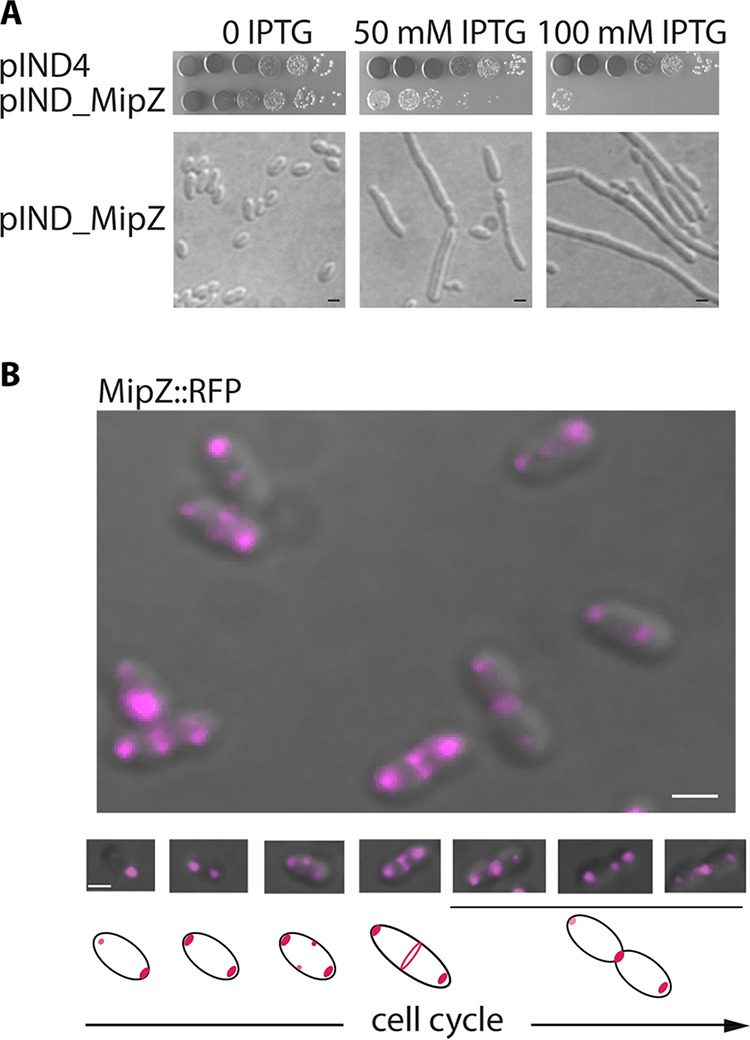
MipZ localization and overexpression. (A) MipZ overproduction phenotype. Levels of viability of cells ectopically producing MipZ are represented in the top panel. Morphological changes induced by MipZ overproduction are represented in the bottom panel. Scale bars, 1 μm. (B) MipZ localization. MipZ-RFP was expressed from the *mipZ* promoter at the *mipZ* locus on the chromosome. The top panel shows an example of representative cells (overlay image: DIC in gray, RFP fluorescence in magenta), and the bottom panel consists of illustrations representing the changes in the position of MipZ during the cell cycle (cells arranged by the size and the advancement of the septation). Scale bars, 1 μm.

A search for division regulators in R. sphaeroides identified a protein with 44% sequence identity to C. crescentus MipZ (17) (EGJ22543.1; see Fig. S1 in the supplemental material). Deletion of the *mipZ* gene was possible only when an extra copy was present on the chromosome, indicating that the *mipZ* gene product is essential. Increased production of MipZ led to minicell formation, filamentation, and cell death, suggesting that MipZ is a negative regulator of cell division in R. sphaeroides (Fig. 1A).

10.1128/mBio.02515-18.1 FIG S1 Rhodobacter sphaeroides MipZ sequence analysis. (A) Amino acid (aa) sequence alignment. The Caulobacter crescentus MipZ sequence (gi|13423660|gb|AAK24136.1| MipZ [Caulobacter crescentus *CB15*]) and the Rhodobacter sphaeroides MipZ candidate sequence (gi|332277228|gb|EGJ22543.1| MipZ [Rhodobacter sphaeroides *WS8N*]) were aligned using Culstal2.1. Multiple-sequence alignment and amino acid colors and conservation (asterisks and dots) follow the Clustal code. The G12, K16, and D40 amino acids mutated in the study are highlighted by a red star on the R. sphaeroides MipZ sequence (amino acids highlighted in red correspond to divergence in primary amino acid sequence and 3D structure between Rhodobacter sphaeroides and Caulobacter crescentus MipZ proteins analyzed as described for panel B). (B) Rhodobacter sphaeroides MipZ dimer 3D structural conformation modeling. Swiss-Model was used to find the best evolutionarily related 3D structure matching the target R. sphaeroides MipZ structure (EGJ22543.1) in the Swiss-Model template library with BLAST (45) and HHBlits (32). C. crescentus MipZ monomer (2xj4.1.A) and dimer (2xj9.1.A) locked forms were the best match (global model quality estimation [GMQE] values of 0.75 and 0.73, respectively, and qualitative model energy analysis [QMEAN] values of −1.42 and −2.66). The blue to red color gradient shows the degrees of confidence (high to low) in the modeling of the MipZ dimer 3D structural conformation based on 2xj9.1. Low-confidence reconstruction in externally exposed loops of the protein corresponds to protein domains with a low degree of amino acid conservation (highlighted in red in panel A). (C) Phylogenetic tree of ParA, MinD, and MipZ proteins constructed using Phylogeny.fr (46). The MinD representatives are shaded in green, and the MipZ representatives are shaded in purple. Download FIG S1, TIF file, 12.1 MB. Copyright © 2019 Dubarry et al. 2019 Dubarry et al. This content is distributed under the terms of the Creative Commons Attribution 4.0 International license.

We replaced *mipZ* in the genome with *mipZ-RFP* (*mipZ*-red fluorescent protein gene) expressed in the native locus behind the native promoter (Fig. 1B). MipZ-RFP is functional, with less than 0.5% of population forming minicells. MipZ-RFP exhibits a complex and dynamic localization pattern occupying three positions in the cell, including both poles and the septum. Classifying the cells by size, as a measure of cell age, revealed that MipZ forms a focus at the new pole in newly divided cells; as the cell grows, MipZ forms a focus at both poles and at midcell, with the levels of fluorescent intensity changing at all three sites; as division progresses, the MipZ present at the septum forms a clear ring which concentrates to a bright focus as septation continues (Fig. 1B).

The striking observation of MipZ at the septum as well as at the polar zones indicates a more complex role in cell division than has been shown for C. crescentus. We therefore investigated the possible roles of MipZ at both the poles and midcell.

### Choreography of *OriC1* and *OriC2*.

**FIG 2:**
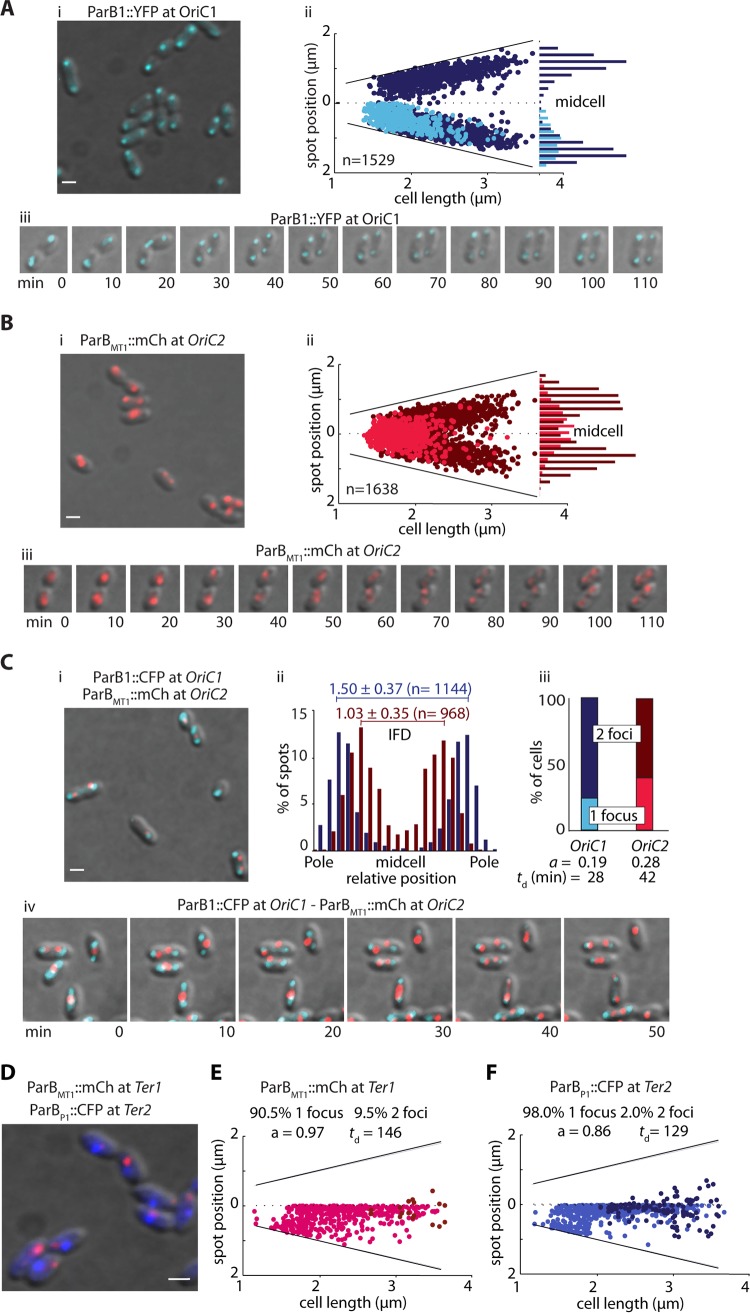
*OriC* and *Ter* dynamics. (A and B) *OriC1* localization by ParB1-YFP (A) and *OriC2* localization by the ParB_MT1_-mCherry/*parS*_MT1_ system (B) (*n*, number of cells analyzed). For each, panel i shows a snapshot of representative cells and panel ii shows the positions of one focus (light color) or two foci (dark color) in the cells. (iii) Dynamic of OriC foci throughout the cell cycle (*n*, number of cells analyzed). (C) OriC1 and OriC2 colocalization. (i) Snapshot of representative cells. OriC1 is localized by ParB1-CFP (in cyan) and *OriC2* by the ParB_MT1_-mCherry/*parS_MT1_* system (in red). (ii) Relative positions of the foci in the cells with 2 foci, with the average interfocal distance (IFD) indicated in micrometers. (iii) Percentages of cells with 1 and 2 foci. The timing of *OriC1* and *OriC2* duplication (*t_d_*) was estimated from the 150-min generation time and the age of the cell (*a* values were between 0 and 1 and were determined using the formula *a* = −[ln(1 − *F*/2)]/ln(2) where *F* represents the fraction of cells with 1 focus) (31). (iv) Dynamics of *OriC* foci throughout the cell cycle. (D) *Ter1* and *Ter2* colocalization images generated using ParB_MT1_-mCherry/*parS_MT1_* (red) and *Ter2* localization by ParBP1-CFP/*parS_P1_* (blue). *n* = 808. (E and F) *Ter1* localization pattern (E) and *Ter2* localization pattern (F). Positions corresponding to one focus (light color) or two foci (dark color) in the cells are indicated. The percentages of cells with 1 focus and percentages of cells with 2 foci are shown on each graph as well as the cell age (*a*) and the duplication timing in minutes (*t_d_*). Scale bars, 1 μm.

In C. crescentus, MipZ links the inhibition of FtsZ polymerization at the poles to the segregation of chromosome by directly interacting with nucleoprotein complex ParB/*parS* at *OriC*. As MipZ is also at a pole in R. sphaeroides, we hypothesized that it might link cell division to the segregation of one or both chromosomes in R. sphaeroides. We therefore investigated the localization of the *Ori* sites of both C1 and C2 (*OriC1* and *OriC2*) in living cells (Fig. 2).

We identified the origin region of each chromosome by cumulative GC skew analysis and searched for replication and partition loci (Fig. S2). To localize the origins of replication in live cells, we took advantage of the *parABS* system on each chromosome and of the findings that ParB proteins colocalize with *OriC* through their binding to *parS* sequences (26–28) and that each *parABS* system is specific to the replicon that encodes it (20).

10.1128/mBio.02515-18.2 FIG S2 Characterization of OriC and Ter regions of Rhodobacter sphaeroides WS8N chromosomes. We identified the origin region (*OriC*) and the terminus region (*Ter*) of each chromosome of R. sphaeroides *WS8N* through the analysis of their DNA sequences (C1, GI 332561612; C2, GI 332561616) for cumulative GC skew (GenSkew software), replication and partition genes and loci (DoriC [45], BLAST, and pBLAST), *dif* site (32), and the inversion of the KOPS sequences (GGNAGGG was used as the KOPS consensus sequence [47] in Clone Manager). The organization of the genome of R. sphaeroides has the same features as that of other multipartite genomes studied: a main chromosome with a classic organization of replication and partition regions at *OriC* (*dnaA* gene, DnaA, boxes, and *parABS* system) and a secondary replicon carrying plasmidic features (here, *parAB* and *repC* genes homologous to the *repABC* cassette carried by the plasmid of the alphaproteobacterium). (A) Description of *OriC1* and *Ter1* loci. Coordinates: *dnaA*, bp 2530136 to 2531135; *OriC1* minimum GC skew, represented as a star, 2439004 bp (*OriC1* defined by DoriC is represented as an oval; *dnaA* boxes are represented by black squares); *parS1* candidates (in light blue), bp 2419151 to 2419166 and bp 2421053 to 2421068; *gidA*, bp 2420988 to 2419195; *gidB*, bp 2419195 to 2418578; *parA1*, bp 2418585 to 2417761; *parB1*, bp 2417761 to 2416859; *Ter1* (represented by a black rectangle) maximum GC skew, bp 856948; *dif1* (represented by a cross in a rectangle), bp 859693 to 859718; *parS_MT1_* insertion to localize *Ter1* locus (represented by a red triangle), bp 868868. (B) Description of *OriC2* and *Ter2* loci. Coordinates: *OriC2* minimum GC skew, represented as a star, bp 183921 (*OriC1* defined by DoriC is represented as an oval; *dnaA* boxes are represented by black squares); *parA*/*repA*, bp 184484 to 183078; *parB*/*repB*, bp 183100 to 182015; *parS2* candidate (in red), bp 182019 to 182042; parS2* candidate, bp 183023 to 183040; parS2** candidate, bp 184601 to 184616; *repC*, bp 185080 to 186393; *parS_MT1_* insertion to localize *OriC2* (represented as a red triangle), bp 178085; *Ter2* (represented by a black rectangle) maximum GC skew, bp 647593; *dif2* (represented by a cross in a rectangle), bp 562797 to 562821; *parS_MT1_* insertion to localize *Ter2* locus (represented as a blue triangle), bp 581700. The *parS2* candidates were defined using the following criteria: a perfect (*parS2*) or nearly perfect (parS2* and parS2**) palindromic sequence of a minimum of 14 bp, present exclusively on the C2 and in the *OriC* region, as the consensus for the *parS* sequences of the *repABC* plasmids of the alphaproteobacterium (GTTnnnnGCnnnnAAC) (48) was not found. The proposed sequences fit with the *parS* consensus (GTTnnnnCGnnnnAAC) sequence present on single chromosomes (universal *parS*) and on the secondary chromosomes of the *Burkholderiales* and the *Ralstoniales* (49). Download FIG S2, TIF file, 9.9 MB. Copyright © 2019 Dubarry et al. 2019 Dubarry et al. This content is distributed under the terms of the Creative Commons Attribution 4.0 International license.

Using ParB1-YFP (ParB1-yellow fluorescent protein), we observed either one or two defined foci in every cell, showing that the replication cycles did not overlap (Fig. 2A, panel i). In newborn cells, *OriC1* localized close to the old pole. *OriC1* then duplicated, and one focus segregated to the opposite pole, resulting in two foci occupying the positions at about 15% and 85% of the cell length (Fig. 2A, panel ii). Comparing *OriC1* localization with that of a polar protein, membrane chemotaxis receptor McpJ (Fig. S3A), confirmed that *OriC1* showed subpolar localization rather than being strictly polar, a finding also reflected by the lesser distances between the sister foci (Fig. S3B). Transient tight polar localization is seen only just after *OriC1* duplication.

10.1128/mBio.02515-18.3 FIG S3 Comparison of ParB1 and MCPJ polar localizations. (A) Localization of MCPJ-GFP (MCPJ-green fluorescent protein) (in green) and ParB1-YFP (ParB1-yellow fluorescent protein) (in cyan) in cells carrying 2 foci. Representative microscopy images are shown. Scale bars, 1 μm. (B) Distance between the two foci (Interfocal distance) of MCPJ and ParB1. MCPJ localization as a function of the cell size forms a linear pattern, reflecting the anchoring of the proteins at the poles, whereas the ParB1 focus distribution shows a shift in cells larger than 2.5 μm. MCPJ localization, *n* = 486 cells; ParB1 localization, *n* = 513 cells. Download FIG S3, TIF file, 5.1 MB. Copyright © 2019 Dubarry et al. 2019 Dubarry et al. This content is distributed under the terms of the Creative Commons Attribution 4.0 International license.

*OriC1* positioning therefore showed 3 phases: in newly divided cells, *OriC1* was loosely localized on one side of old pole; in small (1.7 to 2.5 μm) cells with 2 foci, the *OriC1* foci moved to be very close to the cell poles; in cells above 2.5 μm in size, the positioning was again relaxed (Fig. 2A, panel iii). This move from tightly polar to relaxed localization occurred in cells where the FtsZ ring was positioned at midcell (Fig. S4A) and began constriction (Fig. S4B). This suggests a transient attachment of *OriC1* to a polar factor soon after segregation, which remains until after the reorganization of the chromosomes.

10.1128/mBio.02515-18.4 FIG S4 FtsZ localization and dynamics. FtsZ-YFP is expressed from the leakage of the *Plac* promoter on plasmid pIND4 in the *WS8N* WT strain. (A) Localization of FtsZ present as a single focus at the membrane (light green) or as a ring (dark green). (B) Size of FtsZ rings as a function of the cell size. Two phases can be observed: the FtsZ ring formation phase, where the FtsZ ring diameter is constant (yellow section), and the constriction phase, where the FtsZ ring diameter decreases (orange section). Download FIG S4, TIF file, 3.9 MB. Copyright © 2019 Dubarry et al. 2019 Dubarry et al. This content is distributed under the terms of the Creative Commons Attribution 4.0 International license.

Consistent with this model, simultaneous imaging of ParB1-YFP and mCh-ParA1 (Fig. S5A) revealed that ParB1-*OriC1* complex duplication occurred in a manner concomitant with relocalization of ParA1. In newborn cells, ParA1 appears as a diffuse cloud at the new pole, a fraction of which then condenses into foci that colocalize with the newly segregated sister ParB1s (Fig. S5B).

10.1128/mBio.02515-18.5 FIG S5 Simultaneous visualization of ParA1 and ParB. (A) Representative snapshot of cells producing mCherry-ParA1 (magenta) from the native locus and ParB1-YFP (cyan) from pIND4. Scale bars, 1 μm. (B) Time-lapse imaging of mCherry-ParA1 and ParB1-YFP, illustrating two forms of dynamic behavior. (C) Corresponding kymograph. Cell 1 exhibits dynamic behavior typical of ParA1, which is diffuse at the division pole and then exhibits partial colocalization with newly duplicated sister ParB1 cells. In cell 2, ParB1-*OriC1* exhibits relaxed dynamics as it migrates from pole to pole before it duplicates and locates at the poles with ParA1. Download FIG S5, TIF file, 13.6 MB. Copyright © 2019 Dubarry et al. 2019 Dubarry et al. This content is distributed under the terms of the Creative Commons Attribution 4.0 International license.

To localize *OriC* of C2 (*OriC2*), we used the ParB^MT1^/*parS* system (Fig. S2B) developed in E. coli (29) as all attempts to conjugate a plasmid coding for a ParB2 fusion protein in the R. sphaeroides wild-type (WT) strain failed. *OriC2* localized at midcell in 1-focus cells, and after duplication and symmetric segregation, the *OriC2* foci localized at about 30% and 70% of the cell length (Fig. 2B). Interestingly, this pattern is more similar to that identified in the unrelated gammaproteobacterium Vibrio cholerae (19, 30) than to that recently described in the alphaproteobacteria Sinorhizobium meliloti (22) and Brucella abortus (31).

Following the two loci simultaneously (Fig. 2C, panel i) and calculating the average interfocal distance (IFD) in the population of cells with 2 foci for each origin confirmed their different positions in the cell (Fig. 2C, panel ii). Interestingly, the comparison of the percentage of cells with 1 focus to the percentage of cells with 2 foci for each locus (25% to 75% versus 35% to 65%) revealed a difference between their spatial separation times (Fig. 2C, panel iii). By calculating the age of the cell at the time that the focus duplicated (31) to a 150-min cell cycle (Fig. 2C, panel iii), we estimated duplications of *OriC1* foci at ∼28.5 min of the cell cycle and of *OriC2* foci at ∼42 min. The earlier separation of *OriC1* sister foci was observed in real time in the time-lapse experiments (Fig. 2C, panel iv) and was further confirmed in cephalexin-treated filamentous cells (Fig. S6A and B). It is noteworthy that in all multichromosomic bacteria studied so far, the origin of the biggest chromosome is always the first to be segregated.

10.1128/mBio.02515-18.6 FIG S6 Localization of *OriC* and *Ter* in cephalexin-treated cells. (A and B) *OriC1* and *OriC2* colocalization. *OriC1* is localized by the use of ParB1-CFP (in cyan) and *OriC2* by the ParB_MT1_-mCherry/*parS_MT1_* system (in red) in filamentous cells due to constriction inhibition mediated by cephalexin treatment. Scale bars, 1 μm. (C) *Ter1* and *Ter2* colocalization. *Ter1* is localized by ParB_MT1_-mCherry/*parS_MT1_* (red) and *Ter2* by ParBP1-CFP/*parS_P1_* (blue). Scale bars, 1 μm. Download FIG S6, TIF file, 9.5 MB. Copyright © 2019 Dubarry et al. 2019 Dubarry et al. This content is distributed under the terms of the Creative Commons Attribution 4.0 International license.

### Localization of *Ter1* and *Ter2*.

In order to better understand the process of segregation of the two chromosomes, we also characterized the segregation pattern of their terminus regions (*Ter1* and *Ter2*). These were identified using cumulative GC sequence analysis and the positions of putative *dif* sites (32) (Fig. S2). We introduced a *parS*_P1_ sequence and a *parS*_MT1_ sequence into an intergenic region of convergent genes close to the *dif* site on C1 and C2, respectively, and expressed fluorescently labeled ParB_P1_ and ParB_MT1_ from pIND4 to visualize the two loci (Fig. 2D to F).

*Ter1* and *Ter2* are at the new pole in newborn cells, and Ter2 migrates from the pole to midcell before Ter1. Both *Ter* sites remained as single foci until just before the end of the cell cycle (Fig. 2E and F), but Ter2 duplicated before Ter1, showing that the first event and last event of segregation of chromosomes in R. sphaeroides are C1 related. Applying the same method used for *OriC* analysis, we estimated that *Ter2* duplicates at ∼129 min and that *Ter1* duplicates at ∼146 min of a 150-min cycle, giving 117 min between the apparent duplications of *OriC1* and *Ter1* and 87 min between the apparent duplications of *OriC2* and *Ter2*. Interestingly, the time between *Ori* duplication and *Ter* duplication does not reflect the 3-fold difference in size (DNA length) between C1 and C2 replicons. This suggests either a difference in the replication rates or, more likely, a difference in the control of cohesion of sister chromatids of these two chromosomes as suggested by the delay in Ter1 segregation observed in some cephalexin-induced filamentous cells (Fig. S6C). The latter mechanism would ensure the stringent control necessary for synchronizing the segregation of the 2 chromosomes with cell division.

### MipZ and ParB1: localization and dynamic.

**FIG 3:**
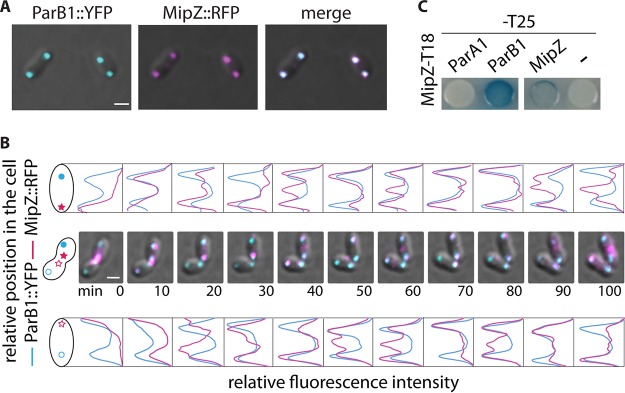
ParB1 and MipZ positions. (A) MipZ-ParB1 colocalization. Representative cells expressing ParB1-YFP (cyan) from the leakage of the *Plac* promoter from pINDParB1-YFP and MipZ-RFP (magenta) from *mipZ* promoter at *mipZ* locus on the chromosome are shown. (B) MipZ-ParB1 dynamics. The upper and lower panels represent the relative fluorescence intensities of ParB1-YFP fluorescent signals (cyan) and MipZ-RFP fluorescent signals (magenta), respectively, observed in the upper and lower cells in the time-lapse experiment represented in the middle area. Fluorescence intensity have been normalized using the lower intensity to subtract the background fluorescence and the higher intensity to normalize the data at a value of 1. For each cell, the new pole is annotated with a star and the old pole with a circle. Scale bars, 1 μm. (C) Bacterial two-hybrid analysis showing MipZ-ParB1 interaction. After IPTG induction, *DHM1* strains containing the appropriate plasmids (pUT18C-MipZ combined with pKT25-ParA1, pKT25-ParB1, pKT25-MipZ, or empty pKT25) were spotted on an X-Gal indicator plate, incubated at 30°C, and inspected for color development.

Given the change in the subpolar position of *OriC1*, we examined whether *OriC1* and MipZ might colocalize by analyzing ParB1-YFP in a strain expressing MipZ*-*RFP from the chromosome (Fig. 3A and B). Colocalization of ParB1 and MipZ was observed at the cell poles throughout the cell cycle, and their direct interaction was confirmed by a bacterial two-hybrid (B2H) assay (Fig. 3C).

This suggests interplay between ParB1, the MipZ position, and the segregation of *OriC1* ensuring coordination of cell division and segregation of C1. As *OriC1* duplicates and segregates before *OriC2* whereas *Ter1* duplicates before *Ter2*, C1 control of MipZ would be sufficient to coordinate duplication and segregation of the complete nucleoid with division.

By following MipZ and ParB1 localization throughout the cell cycle using time-lapse analysis (Fig. 3B), we identified major differences in the positioning pattern compared with C. crescentus (17). We found no evidence for a “tail” or bipolar gradient of MipZ from the cell poles (17) but rather saw symmetric fluorescence intensity profiles for both ParB1 and MipZ foci. Again, unlike C. crescentus, MipZ localized at the old pole with ParB1 in predivision cells and at the new pole of the future daughter cells (Fig. S7). FtsZ is also localized to the new pole immediately after division (33). After duplication of *OriC1* and segregation of one copy to the new pole, MipZ repositioned, showing the same pattern of behavior as the ParB1 focus (Fig. 3B; 0 to 20 min for the top cell and 0 to 30 min for the bottom cell). This suggests that ParB1 repositions MipZ from its earlier postdivision polar location to the DNA/*OriC1* location required for the next round of division.

10.1128/mBio.02515-18.7 FIG S7 ParB1 and MipZ dynamics during the transition from cells in the predivision state to newborn cells. (A to D) Examples of time-lapse microscopy images of cells expressing ParB1-YFP (cyan) from the leakage of the Plac promoter from pINDParB1-YFP and of cells expressing MipZ-RFP (magenta) from the *mipZ* promoter at the *mipZ* locus on the chromosome. Scale bars, 1 μm. Download FIG S7, TIF file, 17.2 MB. Copyright © 2019 Dubarry et al. 2019 Dubarry et al. This content is distributed under the terms of the Creative Commons Attribution 4.0 International license.

### MipZ and FtsZ: localization and dynamic.

Snapshots and time-lapse analysis showed that FtsZ and MipZ colocalized at the septum, with FtsZ being positioned as a ring before MipZ (Fig. 4A and B; 60% of cells with MipZ at the septum versus nearly 80% of cells with FtsZ at the septum). Further time-lapse experiments allowed the direct visualization of the dynamic changes in MipZ localization from one pole to the other pole and to the septum (Fig. 3B [see also Fig. 4C]; images 20 to 50 min).

**FIG 4:**
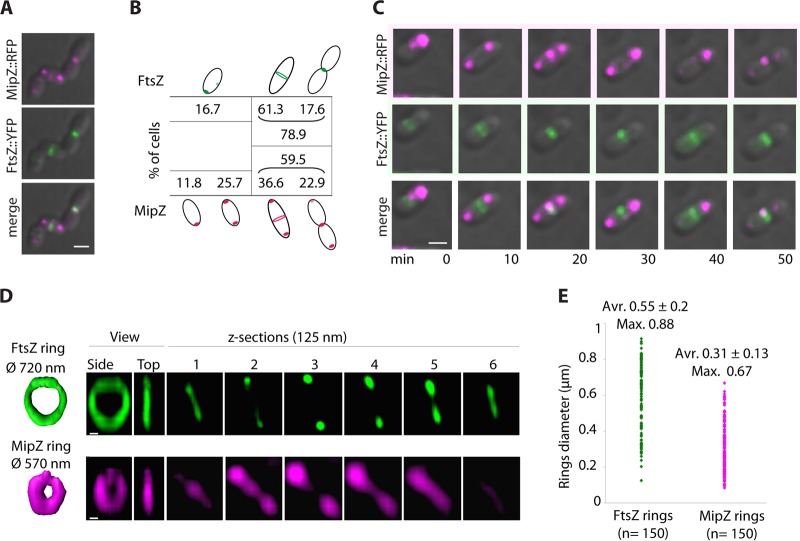
MipZ colocalizes with FtsZ at the septum. (A) MipZ-FtsZ colocalization. Representative cells expressing FtsZ-YFP (green) from the *Plac* promoter leak from pINDFtsZ-YFP and MipZ-RFP (magenta) from the *mipZ* promoter at the *mipZ* locus on the chromosome are shown. Scale bars, 1 μm. (B) MipZ is at the septum after FtsZ. Data represent results of analyses of the cell categories observed as described for panel A. The categories were defined as follows. FtsZ-YFP forms a focus at the new pole or moves toward midcell, FtsZ-YFP forms a ring at midcell (no constriction or early septation), and FtsZ forms a focus at midcell (late septation). A total of 4.4% of the cells showed no defined fluorescent signal. For MipZ localization, the categories were defined as follows. MipZ forms a focus at one pole, MipZ localizes as 2 foci at both poles, MipZ localizes as 2 foci at both poles and as a formation or a complete ring at the septum, and MipZ localizes as 2 foci at both poles and as a focus at the septum. The strain (*WS8N mipZ-rfp* pINDFtsZ-YFP) produces 1.5% long cells due to FtsZ-YFP production and 0.4% minicells. *n* = 459 cells. (C) MipZ-FtsZ dynamics. The images are of representative cells showing FtsZ ring establishment and then MipZ-FtsZ colocalization and dynamics. (D) MipZ forms a unique ring at the septum. 3D-structured illumination microscopy (SIM) was performed for analysis of FtsZ::YFP (top, green) and MipZ::YFP (bottom, magenta). Ring surface representations and side and top view and z-sections (125 nm each) of 3D-SIM reconstructions are shown from left to right. (E) Measurement of diameters of FtsZ rings and MipZ rings from 3D-SIM images. Average (Avr.) values with standard deviations and maximum (Max.)-diameter values show that MipZ rings have a smaller diameter that FtsZ rings.

Simultaneous imaging of FtsZ-YFP and MipZ-YFP using three-dimensional structured illumination microscopy (3D-SIM) (Fig. 4D) showed FtsZ rings similar to those previously observed using other high-resolution microscopy techniques in R. sphaeroides and other bacteria (33–35). In addition, we observe that MipZ-YFP also formed a ring at midcell (Fig. 4D). Measurements of the diameter of FtsZ and MipZ rings showed that, on average, the diameter of the FtsZ rings was larger than that of the MipZ rings (0.55 ± 0.2 μm for the FtsZ rings compared to 0.31 ± 0.13 μm for the MipZ rings) (Fig. 4E). As MipZ is recruited to the septum after FtsZ ring formation, this suggests colocalization during the septation process, possibly regulating FtsZ ring constriction.

### MipZ dimers localize at the septum and inhibit cell division.

**FIG 5:**
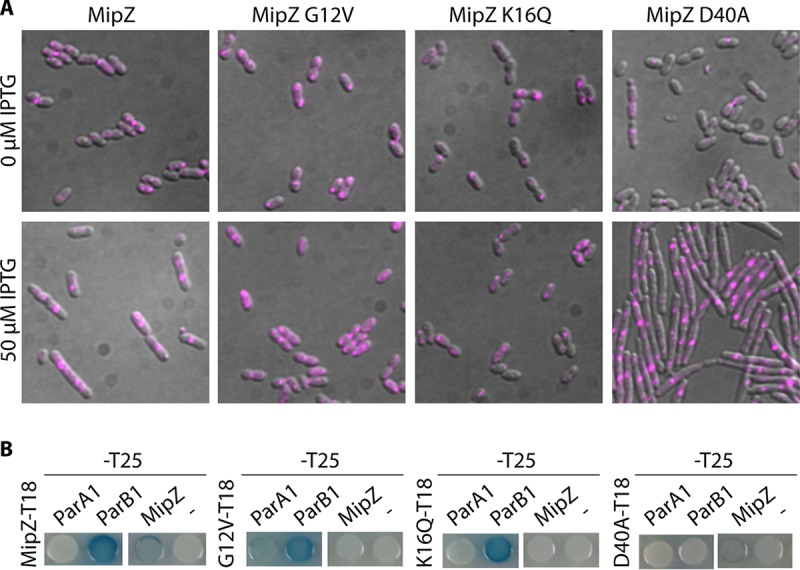
MipZ mutant position, interactions, and phenotypes. (A) MipZ mutant localization. Images show representative cells of mutants MipZ-RFP, MipZ G12V-RFP (mutation in the P-loop, interferes with dimer formation), MipZ K16Q-RFP (mutation in the nucleotide-binding site, reduces affinity for nucleotides), and MipZ D40A-RFP (mutation preventing the catalytic mechanism of ATP hydrolysis) expressed from the Plac promoter leak from pIND4 in WT strain *WS8N* (top panel) and overexpressed by 4 h of IPTG (50 μM) induction (lower panel). Scale bars, 1 μm. (B) MipZ mutant interactions. Bacterial two-hybrid analysis of MipZ mutant interactions with ParA1 and ParB1 was performed.

As MipZ appears to show at least two different behaviors during the cell cycle of R. sphaeroides and because it is related to the ParA family of ATPases, we tested whether the ATP-dependent monomer/dimer switch described for ParA proteins has a role in function (36) by generating a range of mutants in described conserved sites (18, 37, 38). We expressed in the WT strain the predicted monomer locked forms MipZ G12V (mutation in the P-loop, which interferes with dimer formation) and MipZ K16Q (mutation in the nucleotide-binding site, which reduces affinity for nucleotides) and the dimer locked form D40A (ATP hydrolysis-defective mutant), each fused to RFP (Fig. 5A). In parallel, we measured their interaction with ParA1 and ParB1 by B2H assay (Fig. 5B). A clear association of localization and function was observed. The monomer forms were mainly found at the poles, where they interacted with ParB1. They were inactive in division control, as overexpression had no effect on division (Fig. 5A). Interestingly, the MipZG12V monomer, which retains reduced localization at midcell (Fig. 5A), showed a direct interaction with ParA1 (Fig. 5B). This reveals that, in addition to their interaction with ParB, the two ATPases MipZ and ParA1 interact in a nucleotide-binding-dependent manner.

The MipZ dimeric form was exclusively observed at midcell and even low-level expression had a dominant-negative effect on division over the WT proteins, leading to extensive cell filamentation under conditions of overproduction. These data support the hypothesis that MipZ cycles between an ADP-bound monomer and an ATP-bound monomer which interacts with ParAB1 and an ATP-bound dimer which interacts with FtsZ at the septum and controls constriction.

## DISCUSSION

**FIG 6:**
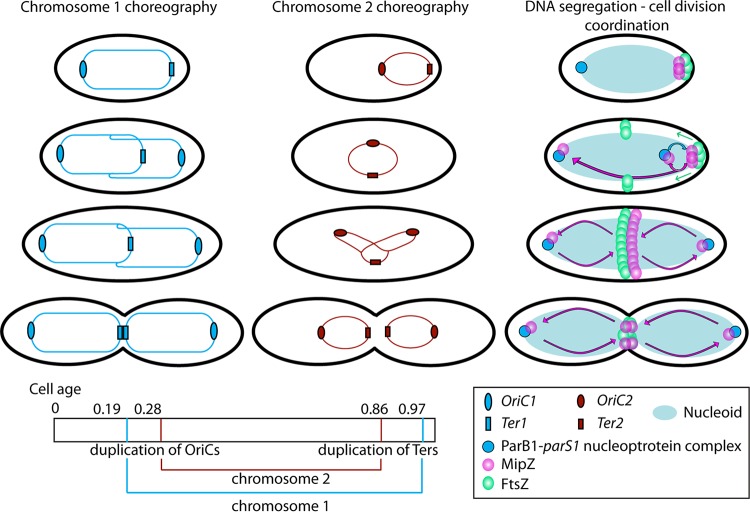
Model of Rhodobacter sphaeroides cell cycle. The two first panels show the choreography of the R. sphaeroides chromosomes. Chromosome 1 follows an asymmetric segregation pattern, with a subpolar localization of its *OriC* regions in a manner that is relaxed in small, ready-to-divide cells and more extensively regulated in long cells. Chromosome 2 follows a symmetric segregation pattern. The ages of the cell at the time of duplication of the *OriC* and *Ter* loci are reported at the bottom (data from Fig. 2). The rightmost panel shows the coordination between the segregation of the chromosomes, represented as a noncompacted nucleoid, and the division process through MipZ. In a newborn cell, MipZ localizes as a focus at the new pole where FtsZ resides and with the ParB1-*parS*-*OriC1* complex at the old pole. After *OriC1* duplication and segregation, ParB relocalizes the MipZ focus from a very polar to a subpolar localization at the new pole and MipZ is observed at ParB at the old pole, finalizing the “ParB catching MipZ” phase. After FtsZ ring initiation at midcell, MipZ colocalizes as a single ring with the division protein, where it stays until the end of the division process. MipZ localization is dynamic and oscillates between the poles, where it interacts with ParB1 as monomers, and the septum, where it acts on the division process as dimers.

**FIG 7:**
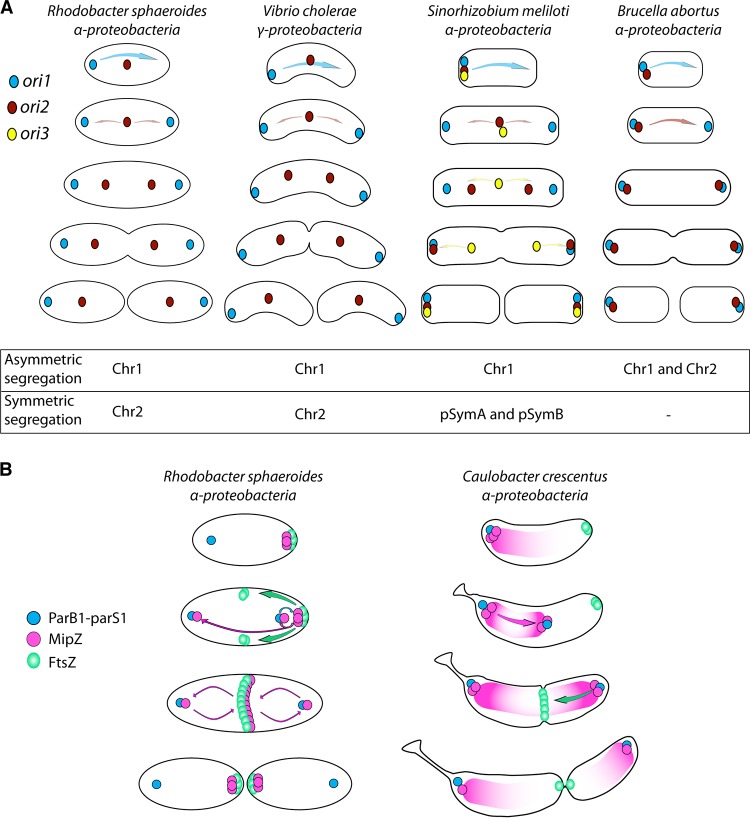
(A) Comparative segregation model for origins of replicons in multichromosomic bacteria. Replicons contained in multichromosomic proteobacteria mainly exhibit two different modes of segregation. Some replicons segregate according to an asymmetric migration from cell pole to cell pole, while others segregate bidirectionally and symmetrically from midcell. Interestingly, various combinations of segregation patterns are observed for the multiple replicons contained in different bacterial species, as show here for Rhodobacter sphaeroides (this work), Vibrio cholerae (21), Sinorhizobium meliloti (22), and Brucella abortus (31). (B) Comparison of protein dynamics through the cell cycle of R. sphaeroides and C. crescentus.

By following the origins and termini of the two chromosomes and the associated controlling proteins ParB and MipZ throughout the cell cycle (Fig. 6), we showed that the dynamic pattern of movement and the roles of the proteins, particularly that of MipZ, are different from those described in related species (Fig. 7).

### Segregation of the R. sphaeroides chromosomes.

The segregation mechanism for the two R. sphaeroides chromosomes appears to be closer to that of the unrelated Vibrio cholerae (30) than to the pattern described for the alphaproteobacteria S. meliloti and A. tumefaciens (19). Unlike the pattern described for V. cholerae or C. crescentus, *OriC1* is only transiently strictly polar, with the predominant subpolar localization more reminiscent of that of Pseudomonas aeruginosa (39). This suggests that the mechanisms of chromosome management and the patterns of segregation are not necessarily confined to closely related species (Fig. 7A).

Analysis of the spatiotemporal patterns of segregation of the *OriC* and *Ter* loci showed no correspondence between the size of the replicons and the timing of their segregation. While the origins replicated and segregated early in the cell cycle, both *Ter* sites showed delays until just before a late stage of septation before segregation occurred, possibly because the last steps of DNA segregation for both chromosomes are controlled by the division process, with cohesion being maintained until late constriction. The data clearly show that the first event and the last event in R. sphaeroides genome segregation correspond to the segregation of the C1, suggesting that this is the “master” chromosome, coordinating genome segregation and cell division.

### MipZ: a new role in cell division control.

R. sphaeroides MipZ shares several characteristics with all the ParA-like proteins, including differential protein-protein interactions and functions controlled by monomer-dimer cycling. Moreover, we observed that, similarly to other ParA-like proteins, MipZ exhibited nonspecific DNA binding activity (see Fig. S8 in the supplemental material). It also shares some of C. crescentus MipZ specificities with MipZ binding to ParB as a monomer and apparently negatively regulates FtsZ polymerization as a dimer. However, the localization and the mechanism of action of the monomer and dimers are very different from those observed in C. crescentus, suggesting that the monomers and dimers regulate chromosome segregation and septation using a mechanism different from those described to date.

10.1128/mBio.02515-18.8 FIG S8 MipZ and ParA1 nonspecific DNA binding. Colocalization of MipZ-YFP and ParA1-YFP with DAPI (4′,6-diamidino-2-phenylindole)-stained DNA in cephalexin-treated E. coli cells is indicated. MipZ-YFP and ParA1-YFP were overproduced from pIND4. Scale bars, 1 μm. Download FIG S8, TIF file, 1.3 MB. Copyright © 2019 Dubarry et al. 2019 Dubarry et al. This content is distributed under the terms of the Creative Commons Attribution 4.0 International license.

The difference in behavior of the two MipZ monomer mutants, with both binding ParB1 but with the nucleotide-binding, nondimer mutant also binding ParA1 and showing localization at midcell (Fig. 5), implies that structural and interaction differences occur as the monomer binds and releases nucleotide, with DNA segregation machine ParABS of C1 probably regulating the dynamics to control cell division. As overproduction of the MipZ dimer inhibits septation, this suggests inhibition of septum formation. However, unlike C. crescentus, there was no observable bipolar gradient, and the dimer never localized to the poles; rather, MipZ dimers formed a ring which colocalized with the FtsZ constriction ring (Fig. 7B). MipZ catalytic domains are well conserved in R. sphaeroides and C. crescentus, while more-variable regions appear notably located on the external surface of the protein (Fig. S1A and B). The differences in the residues exposed at the protein surface could potentially modulate interactions with other factors and explain the singular behavior of MipZ.

We suggest that MipZ monomers and FtsZ monomers in a newly divided cell localize at positions that are very close to the new pole, probably targeted by a polar localizing protein, whereas the *OriC*-*parS* complex is at the other pole with ParB1. *OriC1* duplicates and segregates and ParB1 interacts with MipZ monomers, releasing them and FtsZ from the tight polar position. FtsZ, closely followed by MipZ ATP-bound monomers and dimers, moves to midcell and forms single rings. As both nucleotide-bound monomers and dimers are found at midcell, it is possible that there is a dynamic exchange between the monomers and ring-forming dimers. MipZ clearly oscillates between the poles but does not form a gradient. It seems likely that the movement between the poles and nucleotide binding and exchange with the dimer ring ensure that FtsZ-dependent septation does not happen until the two chromosomes have duplicated and segregated, C2 being controlled by C1, as duplication of both *OriC2* and *Ter2* happens within the time frame of C1 duplication. This unusual pattern of protein movement could represent an adaptation to specific constraints imposed by the multichromosomic architecture of R. sphaeroides genome, or the C. crescentus mechanism may have evolved from a species that buds daughter cells from one pole. The MipZ cycling between two monomer and one dimer forms regulates MipZ dynamics from the poles to the septum and fine-tunes the constriction process in response to segregation of master chromosome 1 through the key coordinator ParAB system.

## MATERIALS AND METHODS

### Bacterial strains, plasmids, and growth conditions.

R. sphaeroides was grown aerobically with shaking at 225 rpm in succinate medium (SUX) (40) or on LB agar plates at 30°C. E. coli was grown aerobically with shaking at 225 rpm in LB medium. Kanamycin (Km), when required, was used at 25 µg/ml. Cephalexin was used at 1 µg/ml and at 10 µg/ml for R. sphaeroides and E. coli, respectively. All expression plasmids derived from pIND4 were conjugated into R. sphaeroides strain WS8N as described previously (41). Expression relied on either promoter leakage or isopropyl β-d-1-thiogalactopyranoside (IPTG) addition. Overproduction experiments were done by spotting of 10 µl of exponential cultures of strain WS8N carrying pIND4 and derivatives on LB agar supplemented of 0, 50, or 100 µM IPTG. Morphological changes induced by MipZ overproduction were visualized after 7 h 30 min (∼5 generations) by microscopy.

### Construction of DNA deletions and insertions and mutants.

DNA insertion or deletion has been performed previously using plasmid pKT18mobsac (42). For *mipZ* deletion, 0.7-kb fragments of upstream and downstream regions of *mipZ* amplified with oligonucleotides delmipZUPa and delmipZUPb and delmipZDWa and delmipZDWb were cloned together into HindIII-EcoR1 in pKT18*mobsac* (pKTdelmipZ), introducing a new Kpn1 site at the junction. This construct allows deletion of 722 of the 810 bp of the gene. *parS* sequences were amplified from pGBKD3-parSP1 and pGBKD3-parSpMT1 and cloned in a unique preexisting NotI or XhoI site or created by overlap PCR in an OriC2, Ter1, or Ter2 DNA fragment cloned in pKT18mobsac. *mipZ* mutants were constructed by PCR mutagenesis. Oligonucleotides with a single point mutation corresponding to the single base change were designed to amplify pINDMipZ-RFP (43) with Phusion DNA polymerase (NEB). PCR were treated with DpnI (NEB), and DH5α cells were transformed. Plasmid DNA was extracted from the resulting clones (MiniPrep kit; Qiagen) and sequenced. Oligonucleotides, plasmids, and strains are listed in Table S1A to C in the supplemental material.

10.1128/mBio.02515-18.9 TABLE S1 Plasmids (A), strains (B), and oligonucleotides (C) used in this work. Download Table S1, DOCX file, 0.04 MB. Copyright © 2019 Dubarry et al. 2019 Dubarry et al. This content is distributed under the terms of the Creative Commons Attribution 4.0 International license.

### Sequence analysis.

Proteins, genes, or sequences of interest were analyzed using pBLAST, BLAST, or Clone Manager software. Sequences of ParA/MinD/MipZ proteins were aligned using ClustalW, and the phylogenetic tree was produced using Phylogeny.fr (http://phylogeny.lirmm.fr/phylo_cgi/index.cgi). The GC skew of each chromosome was calculated and visualized with GenSkew, and the positions of Ori and DnaA boxes were verified with OriFinder.

### Bacterial 2-hybrid assays.

Plasmid pairs encoding the T18 and T25 fusions at the C terminus were cotransformed into DHM1. Several colonies were grown and plated on LB agar containing 50 μg/ml ampicillin (Amp), 25 μg/ml Km, 40 μg/ml X-Gal (5-bromo-4-chloro-3-indolyl-β-d-galactopyranoside), and 250 μM IPTG. Plates were incubated at 30°C overnight and then at room temperature. Color change was inspected after 48 h.

### Fluorescence microscopy and analysis.

Cells were grown to optical density at 550 nm (OD_550_) of 0.2 to 0.4 in SUX medium, washed in SUX, and spread on a layer of SUX medium–1% agarose on a microscope slide.

Differential interference contrast (DIC) and fluorescence images were acquired using a Nikon TE200 microscope equipped with a cooled charge-coupled-device (CCD) camera (Hamamatsu) or a Nikon Eclipse TE2000-U microscope equipped with a Photometrics Cool-Snap HQ CCD camera. Image analysis used ImageJ or MicrobeTracker together with custom programs run in Matlab.

3D structured illumination microscopy (3D-SIM) imaging was performed as described by Lesterlin et al. (44) on an OMX V3 Blaze microscope (Applied Precision/GE Healthcare) equipped with a 60×/1.42 oil UPlanS Apo objective (Olympus) and 488-nm-wavelength-diode lasers and scientific complementary metal-oxide-semiconductor (sCMOS) cameras (PCO). Three-dimensional stacks of FtsZ-YFP and MipZ-YFP were obtained using 8 to 12 125-nm z-sections (resulting from a striped illumination pattern) (angles of −60°, 0°, and +60°) and were shifted in five phase steps. Acquisition settings were 10 ms of exposure for FtsZ-YFP and 35 ms of exposure for MipZ-YFP with 10% and 100% transmission of a 488-nm laser, respectively. Reconstruction was performed using SoftWoRx 6.0 (Applied Precision), and 3D rendering of fluorescent signal together with volume surfacing was done using IMARIS analysis BITPLANE software.
