# Supplementary figures and images for: In Vivo Imaging of the Segregation of the 2 Chromosomes and the Cell Division Proteins of Rhodobacter sphaeroides Reveals an Unexpected Role for MipZ
Source: mBio. 2019 Jan 2;10(1):e02515-18. doi: 10.1128/mBio.02515-18 (PMC6315104; doi:10.1128/mBio.02515-18)

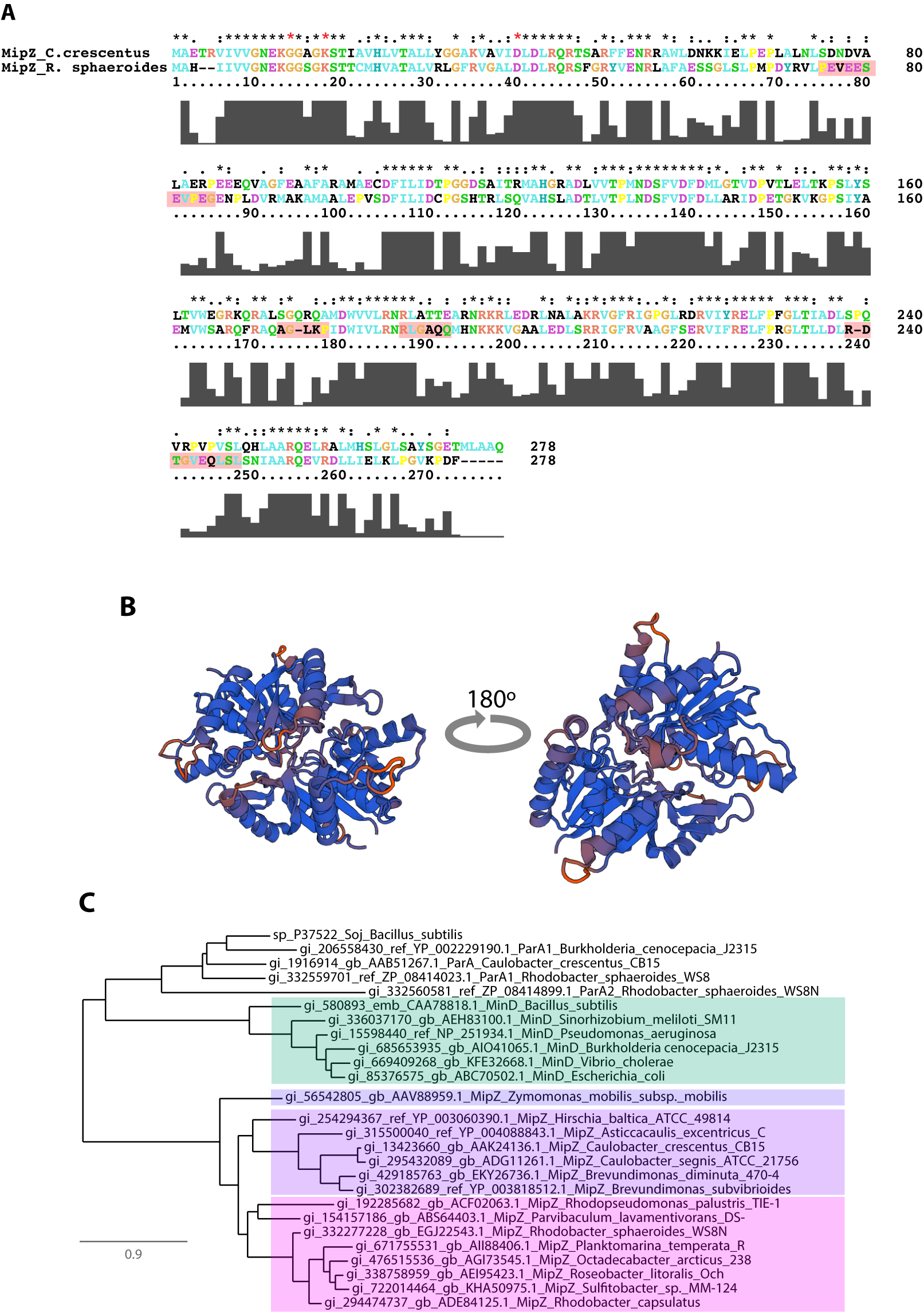

Supplement: FIG S1 [file mbo006184241sf1.tif]

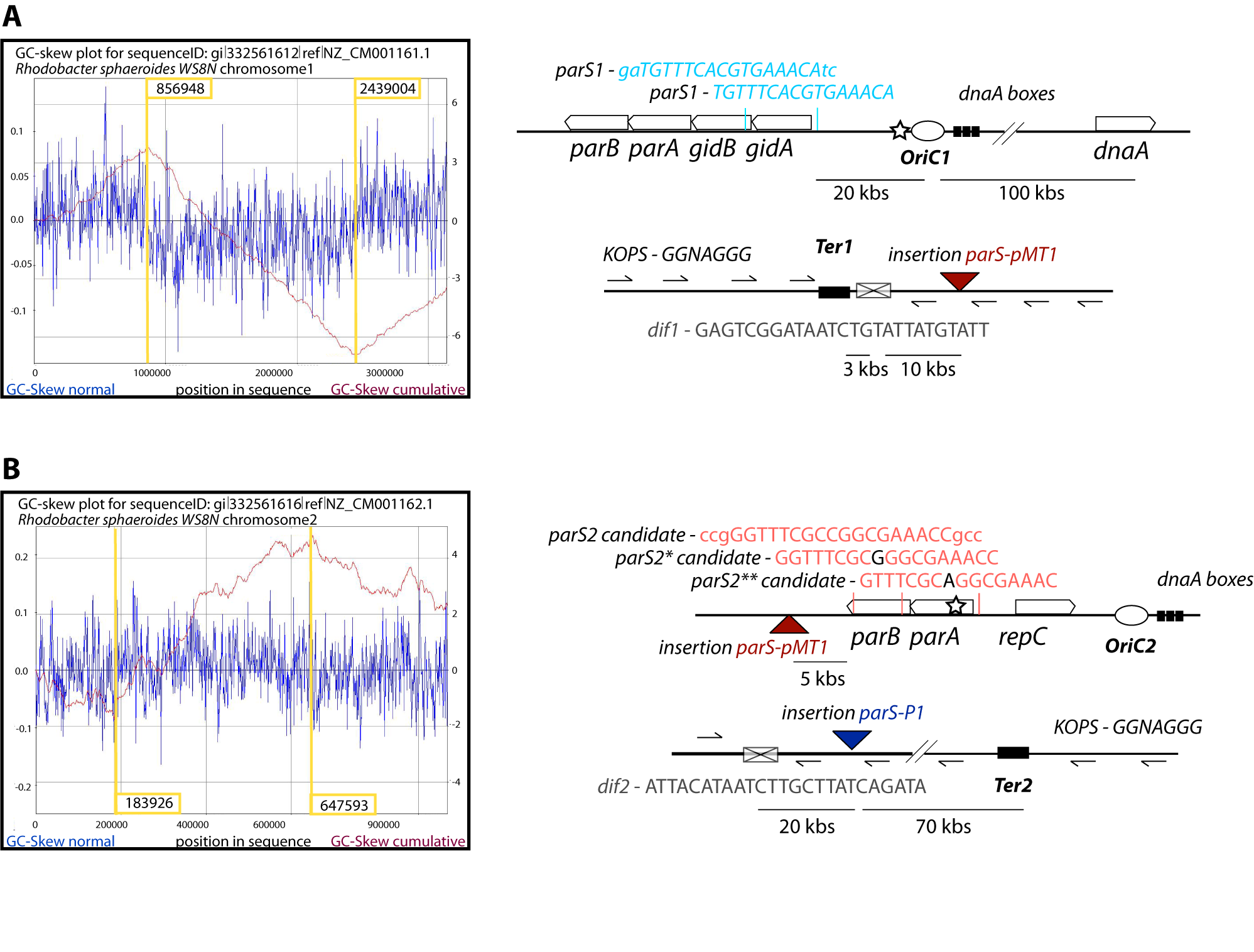

Supplement: FIG S2 [file mbo006184241sf2.tif]

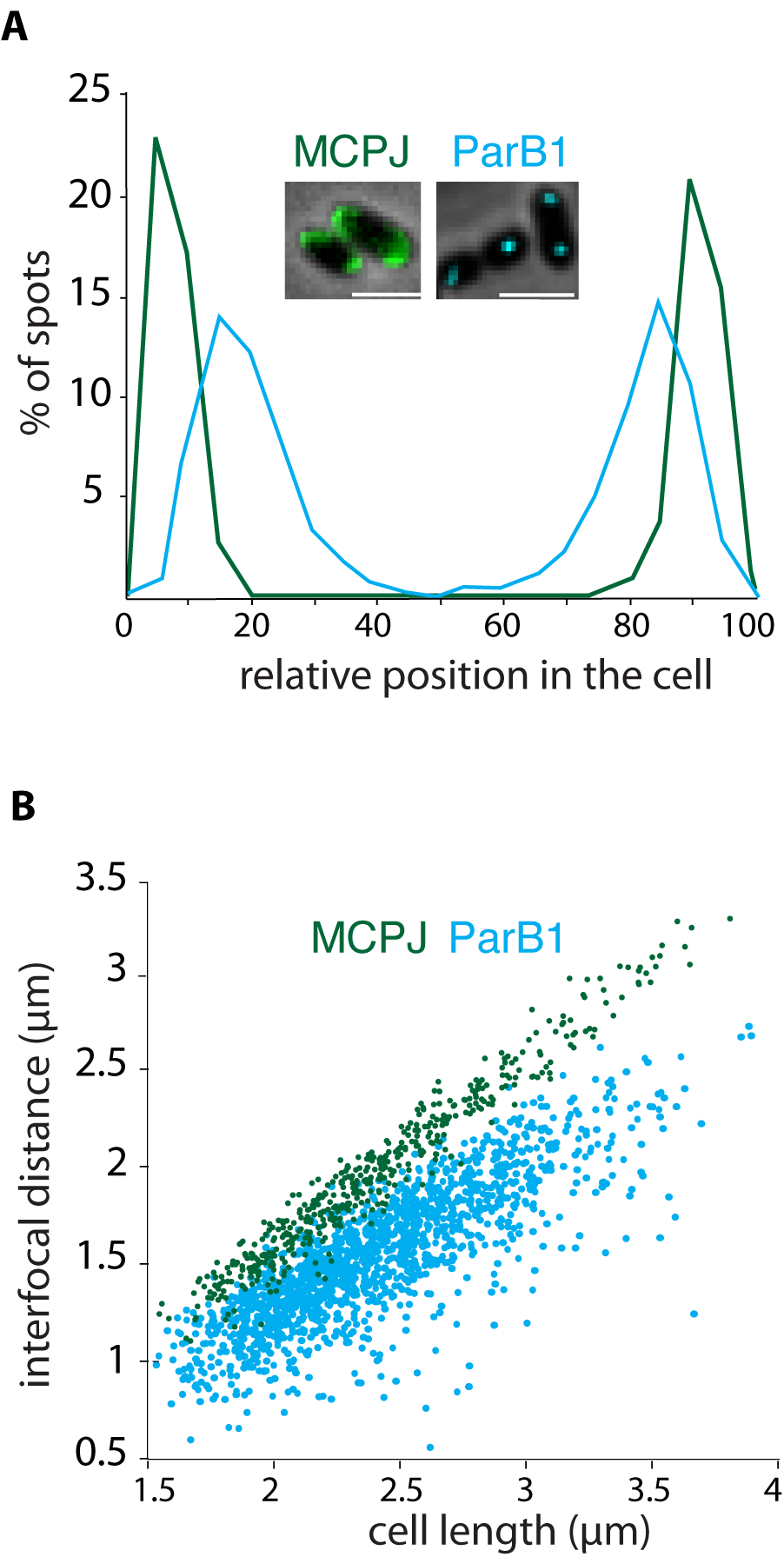

Supplement: FIG S3 [file mbo006184241sf3.tif]

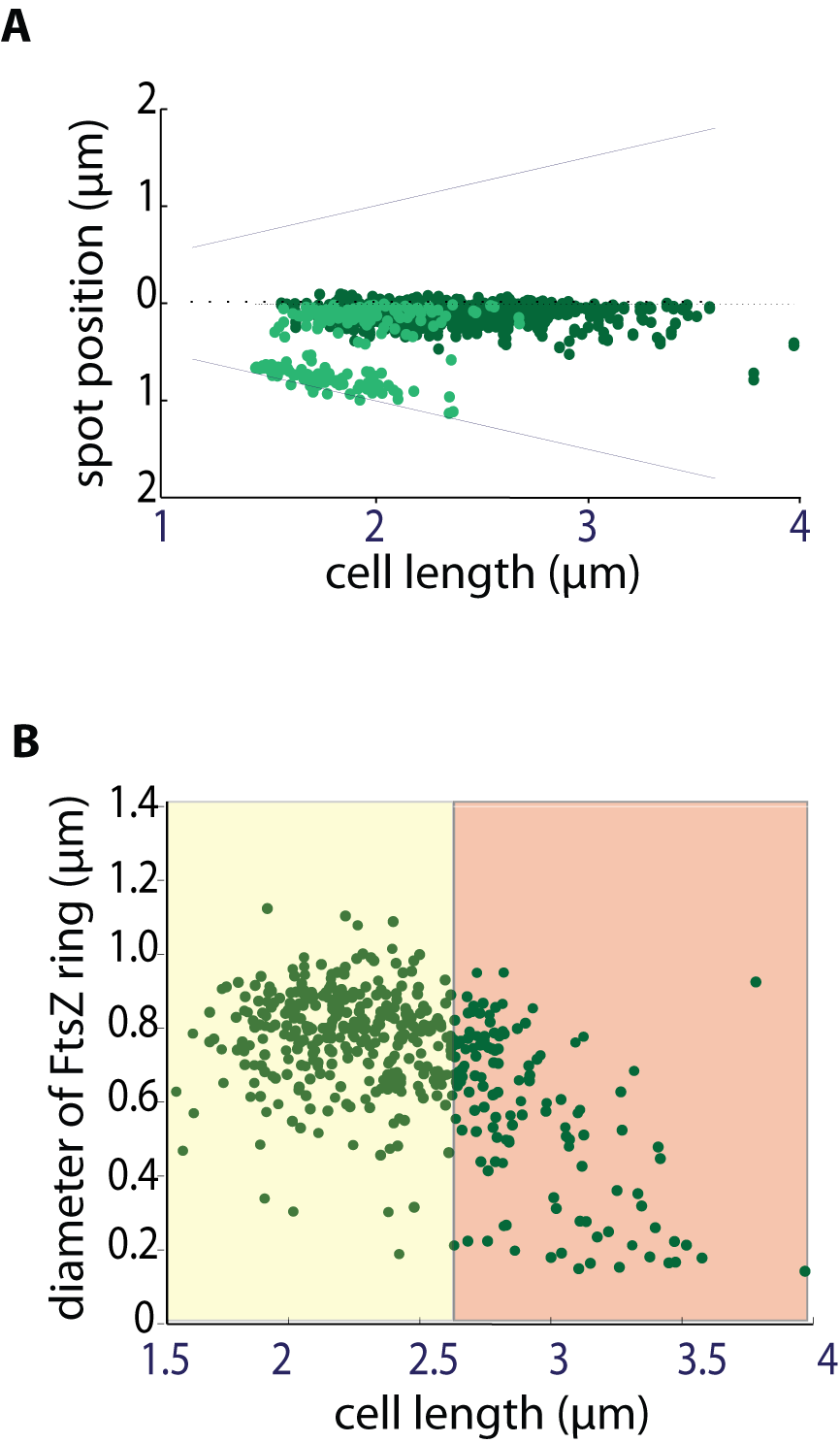

Supplement: FIG S4 [file mbo006184241sf4.tif]

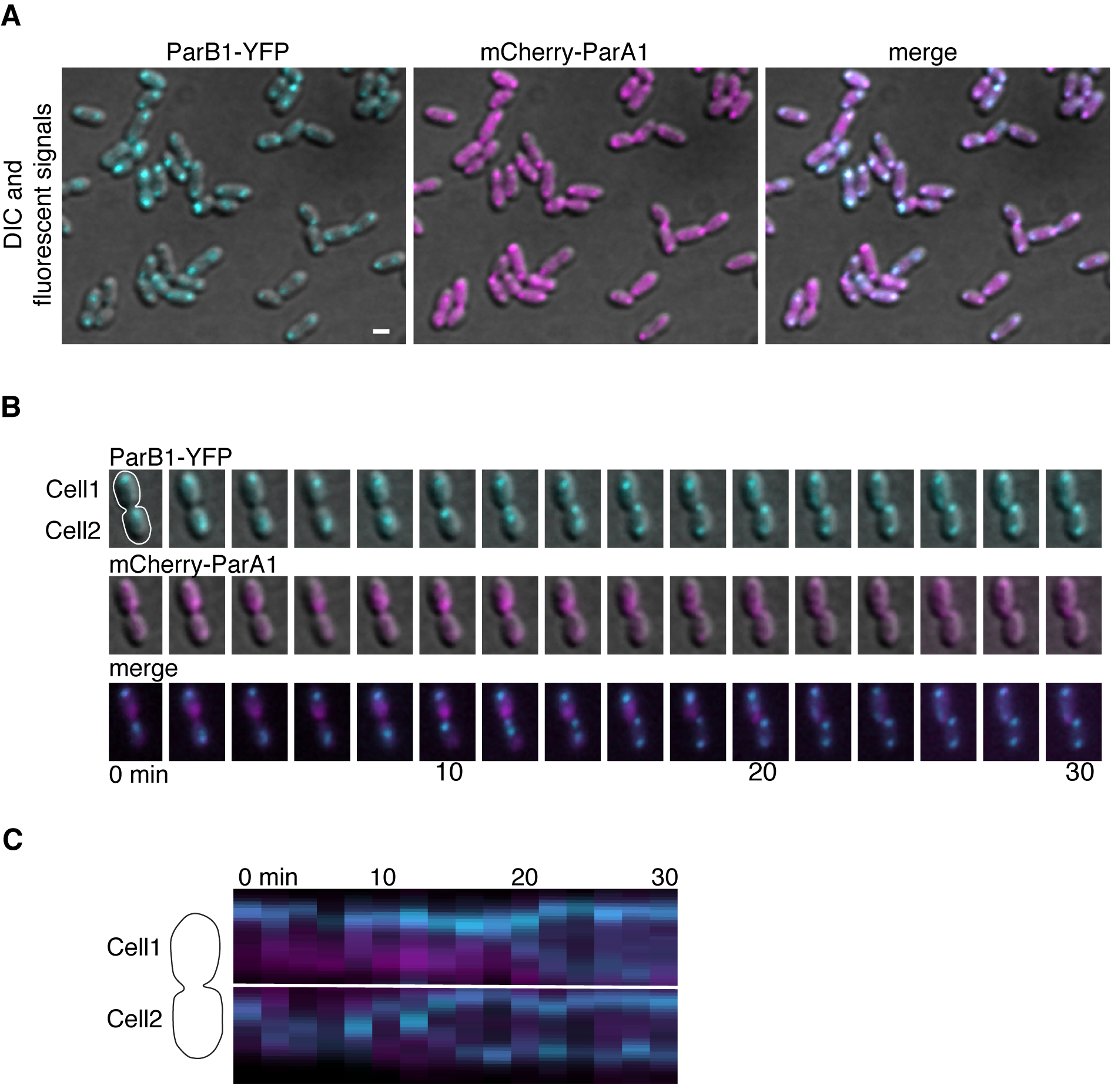

Supplement: FIG S5 [file mbo006184241sf5.tif]

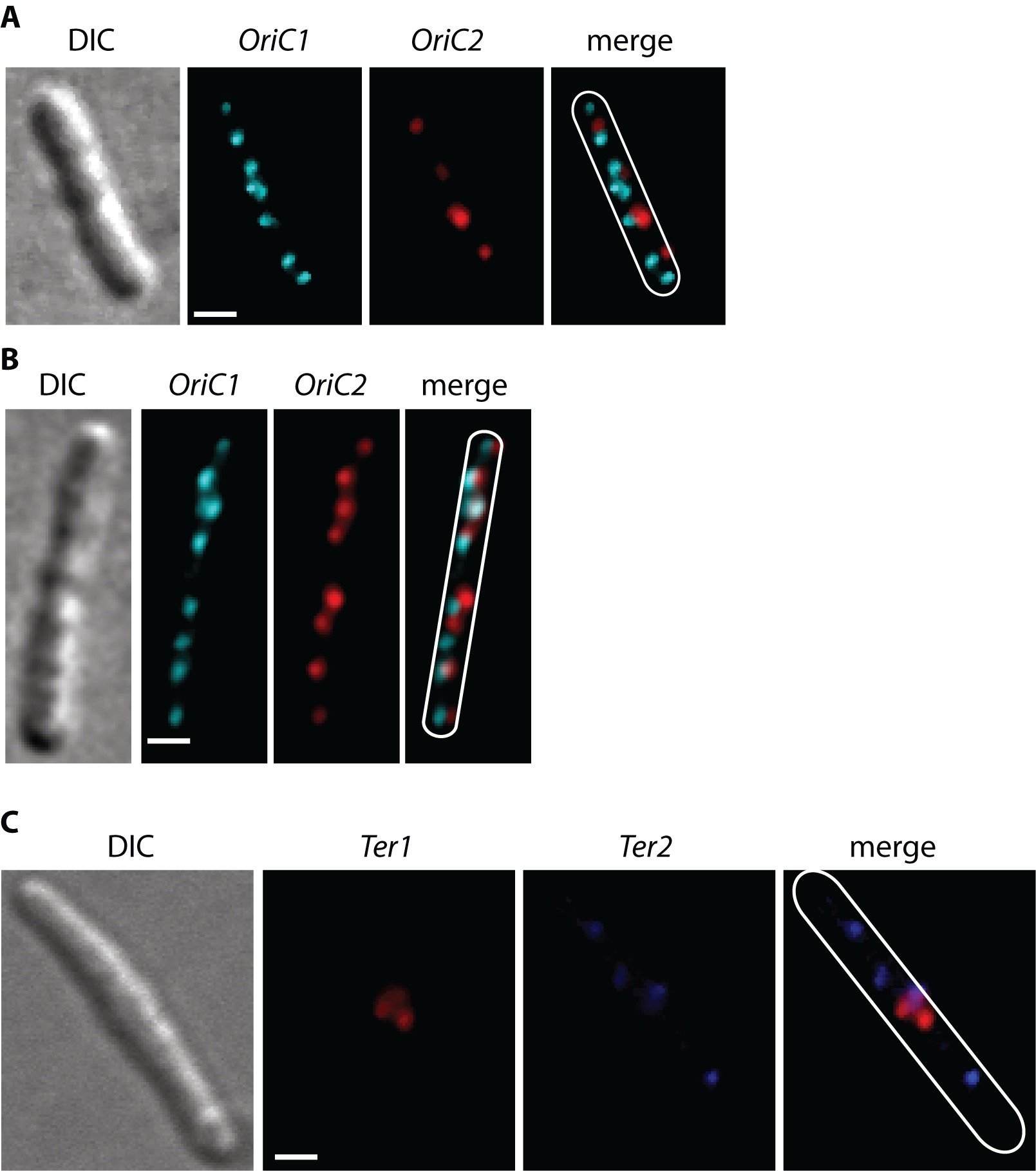

Supplement: FIG S6 [file mbo006184241sf6.tif]

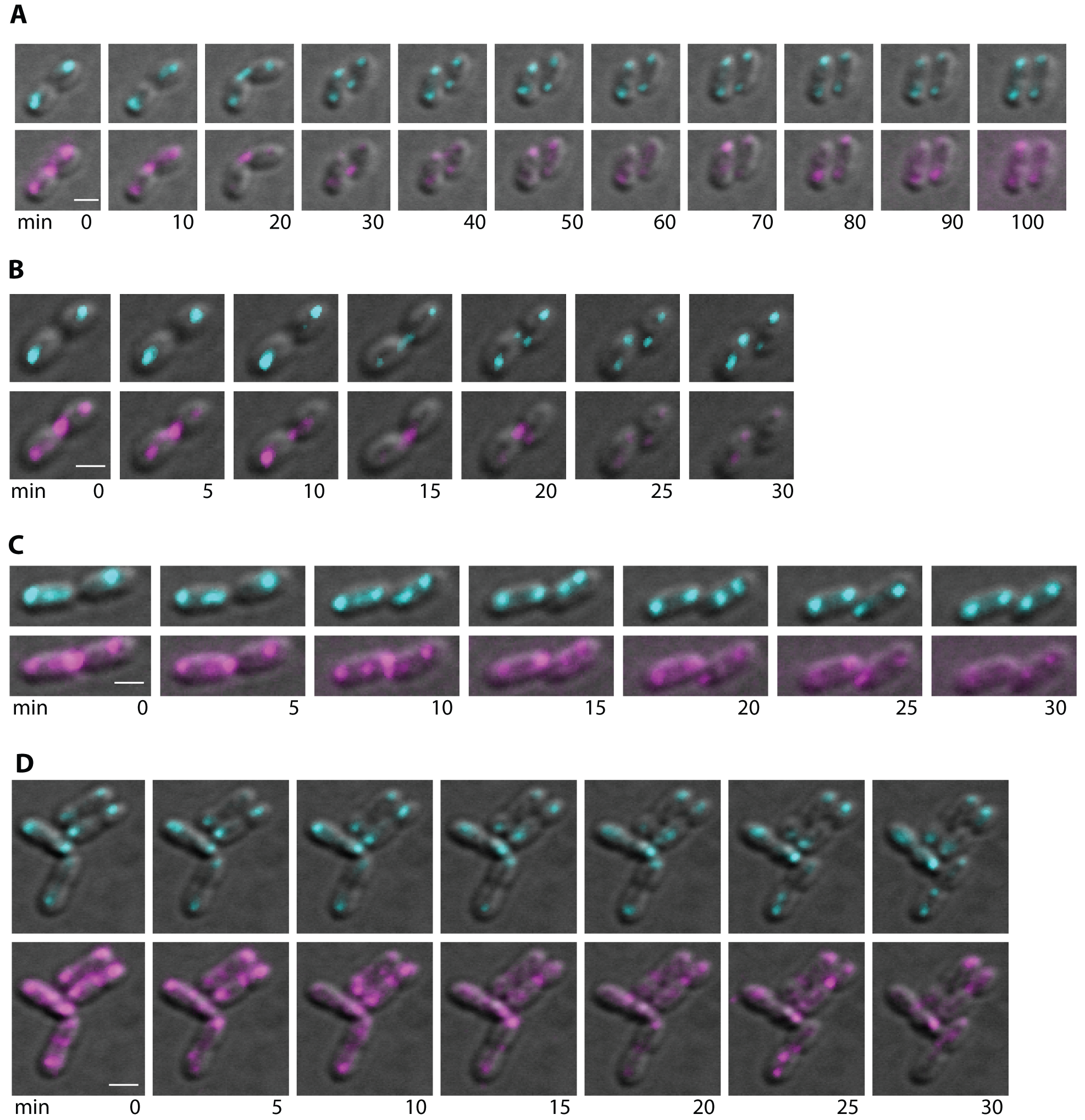

Supplement: FIG S7 [file mbo006184241sf7.tif]

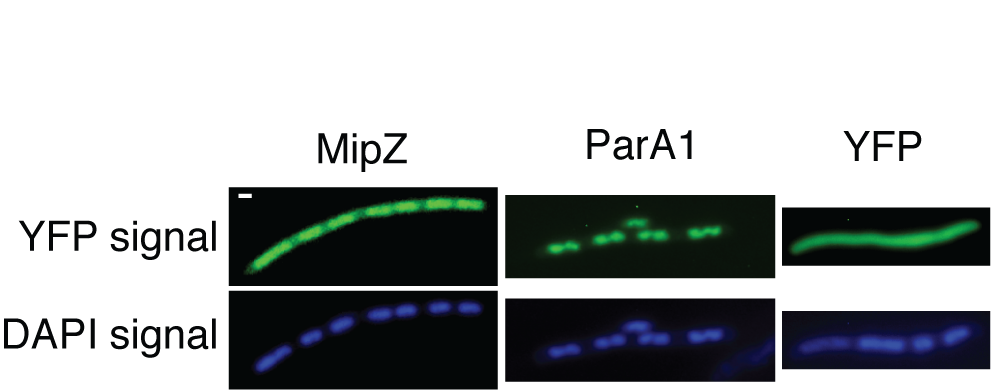

Supplement: FIG S8 [file mbo006184241sf8.tif]
