# Supplementary material for: In Vivo Imaging of the Segregation of the 2 Chromosomes and the Cell Division Proteins of Rhodobacter sphaeroides Reveals an Unexpected Role for MipZ
Source: mBio. 2019 Jan 2;10(1):e02515-18. doi: 10.1128/mBio.02515-18 (PMC6315104; doi:10.1128/mBio.02515-18)
Supplement: TABLE S1 [file mbo006184241st1.docx]

**Choreography of chromosome segregation and cell division proteins in a bacterium with 2 chromosomes reveals an unexpected role for MipZ**

Nelly Dubarry^1,2^, Clare Willis^1,3^,Graeme Ball^1,4^, Christian Lesterlin^1,5^ and Judith P. Armitage^1^*.

**SUPPLEMENTARY MATERIAL**

**Table S1A**

**Table S1B**

**Table S1C**

| **Plasmids** | Description | Construction | Reference |
| --- | --- | --- | --- |
| pIND4 | *R. sphaeroides* expression plasmid. Km resistant. |  | ^26^ |
| pINDYFP | YFP gene on pIND4 – MCS in YFP Cter |  | Laboratory collection |
| pINDCFP | CFP gene on pIND4 – MCS in YFP N or Cter |  | Laboratory collection |
| pINDRFP | RFP gene on pIND4 – MCS in YFP Cter |  | Laboratory collection |
| pINDMipZ | *mipZ* gene on pIND4 under the Plac promoter | *mipZ* amplified with mipZ-5’ and 3’ from WS8N gDNA and cloned in BamH1-BglII into pIND4 | This study |
| pINDMipZ-RFP | RFP gene fused on Cterminal to *mipZ* on pIND4 under the Plac promoter | *mipZ* amplified with mipZ-5’ and mipZ-fus3’ from WS8N gDNA, cloned in BamH1-BglII into pINDRFP | This study |
| pINDMipZ-YFP | YFP gene fused on Cterminal to *mipZ* on pIND4 under the Plac promoter | *mipZ* amplified with mipZ-5’ and mipZ-fus3’ from WS8N gDNA, cloned in BamH1-BglII into pINDYFP | This study |
| pINDParB1-YFP | YFP gene fused on Cterminal to *parB1* on pIND4 under the Plac promoter | *parB1*  amplified with parB1-5’ and parB1-3’ from WS8N gDNA, cloned in NcoI-BamH1 into pINDYFP | This study |
| pINDParB1-CFP | CFP gene fused on Cterminal to *parB1* on pIND4 under the Plac promoter | *parB1*  amplified with parB1-5’ and parB1-3’ from WS8N gDNA, cloned in NcoI-BamH1 into pINDCFP | This study |
| pMS10 | Plasmid pSC101 with *mCherry* fused on N-terminal to *parB_P1_,* Cm resistant |  | ^51^ |
| pMS11 | Plasmid pSC101 with *mCherry* fused on N-terminal to *parB_pMT1_,* Cm resistant |  | ^51^ |
| pINDmCherry-ParB_pMT1_ | *mCherry* fused on N-terminal to *parB_pMT1_* under the Plac promoter | *mCherry-parBpMT1* amplified with Nco1-mCherry and Bgl2-parBpMT1 from pMS11 and cloned in Nco1-BamH1 into pIND4 | This study |
| pINDCFP-ParB_P1_ | CFP fused on N-terminal to *parB_P1_* under the Plac promoter | ParB_P1_ amplified with Nco1-ParBP1 and Bgl2-ParBP1 and cloned in Nco1-Bgl2 into pINDCFP | This study |
| pINDParB1-CFP- mCherry-ParB_pMT1_ | CFP gene fused on C-terminal to *parB1* and *mCherry* fused on N-terminal to *parB_pMT1_* under the Plac promoter | *mCherry-parBpMT1* amplified with Bgl2-mCherry and Bgl2-parBpMT1 from pMS11 and cloned in Bgl2 into pINDParB1-CFP | This study |
| pINDCFP-ParB_P1_-mCherry-ParB_pMT1_ | CFP gene fused on N-terminal to *parB_P1_* and *mCherry* fused on N-terminal to *parB_pMT1_* under the Plac promoter | *mCherry-parBpMT1* amplified with Bgl2-mCherry and Bgl2-parBpMT1 from pMS11 and cloned in Bgl2 into pINDCFP-ParB_P1_ | This study |
| pINDFtsZ-YFP | YFP gene fused on Cterminal to *ftsZ* on pIND4 under the Plac promoter |  | ^37^ |
| pINDMipZG12V-RFP | RFP gene fused on Cterminal to *mipZ* mutant G12V on pIND4 under the Plac promoter | pINDMipZ-RFP amplified with mipZG12V a and b (PCR mutagenesis) | This study |
| pINDMipZK16Q-RFP | RFP gene fused on Cterminal to *mipZ* mutant K16Q on pIND4 under the Plac promoter | pINDMipZ-RFP amplified with mipZK16Q a and b (PCR mutagenesis) | This study |
| pINDMipZD40A-RFP | RFP gene fused on Cterminal to *mipZ* mutant D40A on pIND4 under the Plac promoter | pINDMipZRFP amplified with mipZD40A a and b (PCR mutagenesis) | This study |
| pKT18mobsac | Allelic exchange suicide vector mobilised by *E. coli* S17-1 λ*pir* |  | ^27^ |
| pKT18del*mipZ* | *mipZ* upstream and downstream region cloned into pKT18mobsac | Upstream (UP) and downstream (DW) region of *mipZ* amplified with delmipZUPa and b and delmipZDWa and b from WS8N gDNA were cloned in HindIII – EcoRI into pKT18mobsac. | This study |
| pKT18ins*mipZ-RFP* | *mipZ*-RFP and *mipZ* downstream region cloned into pKT18mobsac | *mipZ-RFP* cloned in HindIII-XbaI into pKT18mobsac as a 1.5 kb fragment digested from pINDMipZ-RFP and downstream region of *mipZ* amplified with hindDWmip1 and 2 and cloned in HindIII as a second step. | This study |
| pGBKD3-parSP1 | *parSP1* sequence in pKD4 plasmid |  | ^52^ |
| pGBKD3-parSpMT1 | *parSpMT1* sequence in pKD4 plasmid |  | ^52^ |
| pKT18*parSpMT1OriC2* | *parSpMT1* sequence cloned in Not1 in OriC2 insertion region | *parSpMT1* sequence amplified with notparSpMT1a and b from pGBKD-parSpMT1 cloned in Not1 in a 1.3 kbs OriC2 fragment amplified by overlap PCR with insC2a, b, c and d to insert a Not1 site from WS8N gDNA and cloned into pKT18mobsac in EcoR1-HindIII. | This study |
| pKT18*parSpMT1Ter1* | *parSpMT1* sequence cloned in NotI in Ter1 insertion region | *parSpMT1* sequence amplified with notparSpMT1a and b from pGBKD-parSpMT1 cloned in Not1 in a 2.9kbs Ter1 fragment amplified with insTer1a and b from WS8N gDNA and cloned into pKT18mobsac in EcoR1-HindIII. | This study |
| pKT18*parSP1Ter2* | *parSP1* sequence cloned in Xho1 in Ter2 insertion region | *parSP1* sequence amplified with xhoparSP1a and b from pGBKD-parSP1 cloned in Xho1 in a 2 kbs Ter2 fragment amplified with insTer2a and b from WS8N gDNA and cloned into pKT18mobsac in EcoR1-HindIII. | This study |
| pKT25 | Two-hybrid vector. Km resistant. |  | ^53^ |
| pUT18C | Two-hybrid vector. Amp resistant. |  | ^53^ |
| pKT25ParA1 | *parA1* cloned into pKT25 | *parA1* amplified with oligonucleotides xbaparA1UP and ecoRIparA1DW from WS8N gDNA and cloned in EcoRI – XbaI into pKT25. | This study |
| pKT25ParB1 | *parB1* cloned into pKT25 | *parB1* amplified with oligonucleotides bamparB1UP and kpnparB1DW from WS8N gDNA and cloned in KpnI – BamH1 into pKT25. | This study |
| pKT25MipZ | *mipZ* cloned into pKT25 | *mipZ* amplified with oligonucleotides bammipZUP and kpnmipZDW from WS8N gDNA and cloned in KpnI – BamH1 into pKT25. | This study |
| pUT18CMipZ | *mipZ* cloned into pUT18C | *mipZ* amplified with oligonucleotides bammipZUP and kpnmipZDW from WS8N gDNA and cloned in KpnI – BamH1 into pUT18C. | This study |
| pUT18CMipZG12V | *mipZG12V* cloned into pUT18C | *mipZG12V*  amplified with oligonucleotides bammipZUP and kpnmipZDW from pINDMipZG12V-RFP and cloned in KpnI – BamH1 into pUT18C. | This study |
| pUT18CMipZK16Q | *mipZK16Q* cloned into pUT18C | *mipZK16Q*  amplified with oligonucleotides bammipZUP and kpnmipZDW from pINDMipZK16Q-RFP and cloned in KpnI – BamH1 into pUT18C. | This study |
| pUT18CMipZD40A | *mipZD40A* cloned into pUT18C | *mipZD40A*  amplified with oligonucleotides bammipZUP and kpnmipZDW from pINDMipZD40A-RFP and cloned in KpnI – BamH1 into pUT18C. | This study |

**Table S1A: Plasmids list**

| **Strain** |  |  |
| --- | --- | --- |
| WS8N | *Rhodobacter sphaeroides* spontaneous nalidixic acid-resistant mutant of wild-type WS8 | ^54^ |
| WS8N*mipZ-rfp* | Replacement of WT *mipZ* gene by *mipZ-rfp* in *Rhodobacter sphaeroides* WS8N strain | This study |
| WS8N*parS*_P1_Ter1*parS*_pMT1_Ter2 | Insertion of *parS_P1_* at Ter1 and *parS_pMT1_* at Ter2 | This study |
| WS8N*mCherry-parA1* | Replacement of WT *parA1* gene by *mCherry-parA1* in *Rhodobacter sphaeroides* WS8N strain | This study |
| *Escherichia coli* DH5α | F– Φ80*lac*ZΔM15 Δ(*lac*ZYA-*arg*F) U169 *rec*A1 *end*A1 *hsd*R17 (rK–, mK+) *pho*A *sup*E44 λ– *thi*-1 *gyr*A96 *rel*A1 | Invitrogen |
| *Escherichia coli* S17-1 λ*pir* | Strain capable of mobilizing the suicide vector pK18mobsacB into *R. sphaeroides*; streptomycin-resistant | ^55^ |
| *Escherichia coli* DMH1 | F^-^*glnV44*(AS) *recA1 endA gyrA96 thi-1 hsdR17 spoT1 rfbD1 cya-854* | ^53^ |

**Table S1B: Strains list**

| **Oligonucleotides** | Sequence |
| --- | --- |
| mipZ-5’ | CAGGGATCCGTGGCGCATATCATCGTGGTGGGCAACGAGAAGGG |
| mipZ-3’ | CATAGATCTGATCAGAAGTCGGGCTTCACCCCCGGCAGCTTCAG |
| mipZ-fus3’ | CAGAGATCTTCCTCCGAAGTCGGGCTTCACCCCCGGCAGCTTCAG |
| parB1-5’ | CAGCCATGGGAGAGAAGAAGATGGAGCGTCG |
| parB1-3’ | CATGGATCCTCCTCCTCCTATGGACCCGTCCCTCGGC |
| Nco1mCherry | CACCCATGGGAAGCAAGGGCGAGGAGGATAACATGGC |
| Bgl2-ParB_pMT1_ | CATAGATCTTCCTCCCTCACCTGATTCTGGAAGTCTTTCCAG |
| Nco1-ParB_P1_ | CATCCATGGGAGTCGAGCAGGTATTCAAGTTATCAACTG |
| Bgl2-ParB_P1_ | CATAGATCTTCCTCCAGGCTTCGGCTTTTTATCGAGGCTC |
| Bgl2-mCherry | CACAGATCTATGAGCAAGGGCGAGGAGGATAACATG |
| mipZG12V a | CGTGGTGGGCAACGAGAAGGCCGGCTCGGGCAAATCCAC |
| mipZG12V b | GTGGATTTGCCCGAGCCGGCCTTCTCGTTGCCCACCACG |
| mipZK16Q a | GAAGGGCGGCTCGGGCCAATCCACCACCTGCATG |
| mipZK16Q b | CATGCAGGTGGTGGATTGGCCCGAGCCGCCCTTC |
| mipZD40A a | GTGGGGGCGCTCGACCTCGCTCTCCGCCAGCGCAG |
| mipZD40A b | CTGCGCTGGCGGAGAGCGAGGTCGAGCGCCCCCAC |
| delmipZUPa | CATGGTACCTTCTCGTTGCCCACCACGATGATATGCGCCACGGGAAG |
| delmipZUPb | CATGAATTCCAACACGATCTCCGCCTCGTTCACCGTGCG |
| delmipZDWa | CATAAGCTTGATCGGCAGCGCATGGATCAGCACGTC |
| delmipZDWb | CATGGTACCGTGCGTGACCTGCTGATCGAGCTGAAGCTG |
| hindDWmip1 | CATAAGCTTAGCGCATGGATCAGCACGTCGCGCTCGGTGAA |
| hindDWmip2 | CATAAGCTTAGGGAGGACCCGATGGCCAATCCGTTG |
| notparSpMT1a | CAAGCGGCCGCAAGGAGGATATTCATATGGACC |
| notparSpMT1b | CAAGCGGCCGCGGTCTGCTATGTGGTGCTATC |
| insTer1a | CGGACCTGGGTCGAATTCACGCTGC |
| insTer1b | CAGAAGCTTCGCGACCTGAGCTTCTCGATCCTC |
| xhoparSP1a | CAGCTCGAGAAGGAGGATATTCATATGGACC |
| xhoparSP1b | CAGCTCGAGGGTCTGCTATGTGGTGCTATC |
| insTer2a | CAGGAATTCCGACCGGGTGGTGCTGCGCTCG |
| insTer2b | CAGAAGCTTTGGCCACGCCGCTCTGGGACAAC |
| insOriC2a | CATAAGCTTGAGACGCTGGTTCTGGTCGATGCGGCC |
| insOriC2b | CAAGACGTCATTCCAAGCGGCCGCGTAACATGAAATCGGTTACAGGC |
| insOriC2c | CGGCCGCTTGGAATGACGTCTTGGTACGACGACTTAGCAGCATCCTCC |
| insOriC2d | CATGAATTCCTTCCAGCAGTTCGGGCTTCAG |
| xbaparA1UP | CATTCTAGAGATGTCTGACTCGAACCGACCCGGAAAGCCGCTC |
| ecoRIparA1DW | CATGAATTCATCACAAGGCGGGTTCCTGTTGGGCCAC |
| bamparB1UP | CATGGATCCCATGGAGAAGAAGATGGAGCGTCGTGGGCTTG |
| kpnparB1DW | CATGGTACCTCATATGGACCCGTCCCTCGGCATGACCG |
| bammipZUP | CATGGATCCCGTGGCGCATATCATCGTGGTGGGCAACGAGAAGGG |
| kpnmipZDW | CATGGTACCTCAGAAGTCGGGCTTCACCCCCGGCAGCTTCAG |

**Table S1C: Oligonucleotides list**
